# Differential expression of the circadian clock network correlates with tumour progression in gliomas

**DOI:** 10.1186/s12920-023-01585-w

**Published:** 2023-07-03

**Authors:** Marina Petkovic, Müge Yalçin, Oliver Heese, Angela Relógio

**Affiliations:** 1 Institute for Theoretical Biology (ITB), Charité-Universitätsmedizin Berlin, corporate member of Freie Universität Berlin, Humboldt-Universität zu Berlin and Berlin Institute of Health, 10117 Berlin, Germany; 2 Molecular Cancer Research Center (MKFZ), Medical Department of Hematology, Oncology, and Tumour Immunology, Charité-Universitätsmedizin Berlin, corporate member of Freie Universität Berlin, Humboldt-Universität zu Berlin and Berlin Institute of Health, 10117 Berlin, Germany; 3 Institute for Systems Medicine, Faculty of Human Medicine, MSH Medical School Hamburg, 20457 Hamburg, Germany; 4 Department of Neurosurgery and Spinal Surgery, HELIOS Medical Center Schwerin, University Campus of MSH Medical School Hamburg, 20457 Hamburg, Germany

**Keywords:** Glioma, Circadian rhythms, Gene expression, Tumor progression, Glioblastoma, Low-grade glioma, Clock-regulated genes, Cancer evolution, Multi-region sequencing

## Abstract

**Background:**

Gliomas are tumours arising mostly from astrocytic or oligodendrocytic precursor cells. These tumours are classified according to the updated WHO classification from 2021 in 4 grades depending on molecular and histopathological criteria. Despite novel multimodal therapeutic approaches, the vast majority of gliomas (WHO grade III and IV) are not curable. The circadian clock is an important regulator of numerous cellular processes and its dysregulation had been found during the progression of many cancers, including gliomas.

**Results:**

In this study, we explore expression patterns of clock-controlled genes in low-grade glioma (LGG) and glioblastoma multiforme (GBM) and show that a set of 45 clock-controlled genes can be used to distinguish GBM from normal tissue. Subsequent analysis identified 17 clock-controlled genes with a significant association with survival. The results point to a loss of correlation strength within elements of the circadian clock network in GBM compared to LGG. We further explored the progression patterns of mutations in LGG and GBM, and showed that tumour suppressor *APC* is lost late both in LGG and GBM. Moreover, *HIF1A*, involved in cellular response to hypoxia, exhibits subclonal losses in LGG, and *TERT*, involved in the formation of telomerase, is lost late in the GBM progression. By examining multi-sample LGG data, we find that the clock-controlled driver genes *APC*, *HIF1A*, *TERT* and *TP53* experience frequent subclonal gains and losses.

**Conclusions:**

Our results show a higher level of disrgulation at the gene expression level in GBM compared to LGG, and indicate an association between the differentially expressed clock-regulated genes and patient survival in both LGG and GBM. By reconstructing the patterns of progression in LGG and GBM, our data reveals the relatively late gains and losses of clock-regulated glioma drivers. Our analysis emphasizes the role of clock-regulated genes in glioma development and progression. Yet, further research is needed to asses their value in the development of new treatments.

**Supplementary Information:**

The online version contains supplementary material available at 10.1186/s12920-023-01585-w.

## Background

Malignant gliomas (WHO grade III and IV [1]) are the most common type of primary brain tumours, representing 2% of all cancer types, 28% of all brain tumours and 80% of all malignant brain tumours [2, 3], with the worldwide age-adjusted incidence of all gliomas ranging from 4.67 to 5.73 per 100 000 individuals [4]. Glial tumours emerge from astrocytes, oligodendrocytes or glial precursor cells [5, 6]. By using molecular markers (e.g., *IDH*, *ATRX*, *1p19q*) and histological characteristics like cellular heterogeneity, nuclear polymorphisms, vascular proliferation and intratumoral necrosis, gliomas can be classified in grade I to IV, reflecting the clinical outcome of the patients [7]. For instance, patients with the most malignant glioma WHO grade IV, called glioblastoma multiforme (GBM), have a median overall survival of 15 months despite multimodal therapies such as surgery, radiation therapy and chemotherapy. [8] [2].

Several single nucleotide polymorphism (SNP) loci have been associated with the increased susceptibility to develop different types of gliomas. For example, the presence of the variant on the 17p13 locus in the tumour suppressor gene *TP53* (tumour protein p53) [9], important in maintaining cellular homeostasis [10], as well as variants of telomerase reverse transcriptase (*TERT)* [11] - important for the telomer maintenance and cell immortalization, a long non-coding RNA *CCDC26* - modulator of differentiation and death, and the tumour suppressors cyclin-dependent kinase inhibitors 2A and 2B (*CDKN2A* and *CDKN2B)* [12], which regulate cell cycle progress, correlate with higher glioma incidence. A SNP near the intron/exon boundary of exon 7 in epidermal growth factor receptor (*EGFR)* – involved in controlling cell proliferation, whose alterations are frequent in different cancer types [13], increases the risk of developing GBM [14]. Variation on the 8q24 locus in a long non-coding RNA *CCDC26* raises the risk of developing oligodendrogliomas and astrocytomas with mutant *IDH1* or *IDH2* [15], and the variant on the 11q23 locus in pleckstrin homology like domain family B member 1 (*PHLDB1)* elevates the chance of developing IDH-mutant gliomas regardless of the glioma histology [16]. Treatment of gliomas is challenging due to high resistance to treatment, high intra-tumour heterogeneity and high recurrence rate. Low grade gliomas (LGG) recur in 28–72% cases [17], and high-grade gliomas in at least 70% of cases after initial treatment [18, 19], with the 5-year survival rates varying between 94.4% for grade I (pilocytic astrocytoma) to 4.7% for GBM [4].

Recent studies have proposed a connection between the onset and the progression of glioma and the circadian clock [20, 21]. A properly functioning circadian clock is crucial for cellular homeostasis, and its dysregulation has been associated with a variety of pathologies, such as sleep disorders [22–24], mood disorders [25, 26], neurogenerative diseases [27–29], and cancer [30]. In mammals, the circadian clock generates endogenous 24-hour rhythms in approximately 40% of all protein-coding genes across different tissues [31], and enables organisms to anticipate and respond timely to external stimuli and adapt behaviour and molecular processes to specific times of the day, for example, to separate incompatible metabolic processes [32, 33]. Therefore, a variety of biological processes including RNA processing [34, 35], metabolism [33, 36], the cell cycle [36], apoptosis [37, 38], immune system regulation [39] and even cellular migration [38] are controlled by the circadian clock. Cellular clocks consist of a set of transcriptional/translational activators and repressors that operate in interlocking feedback loops [40]. The core-clock proteins *BMAL1* and *CLOCK* form heterodimers, bind to the E-box elements in the promoter region of *PER1-3* and *CRY1/2* and initiate their transcription. Upon accumulation, PER and CRY proteins prevent binding of the *BMAL1/CLOCK* complex to E-boxes and inhibit the transcription of *BMAL1/CLOCK* target genes. In addition, *BMAL1/CLOCK* heterodimers activate the transcription of *REV-ERB*s and *ROR*s, which in turn compete for *RORE*s binding sites in the promoter region of *BMAL1* and regulate its transcription negatively (*REV-ERB*s) and positively (*ROR*s). These core-clock elements account for the circadian expression of clock-regulated genes in different tissues [17, 30, 41]. Such genes are referred to as clock-regulated genes. Several core-clock genes have been reported to be dysregulated in gliomas, namely, *CLOCK* [42] and *BMAL1* [43] were found to be overexpressed in high-grade glioma cells and GBM tissue, respectively. *BMAL1* overexpression was also found to inhibit cell invasion in human glioma cell lines [44]. On the contrary, *PER1/2* show lower expression in higher gliomas [45], and *CRY1/2* show lower expression in gliomas compared to normal tissue [46]. Lower *HIF1A* expression under hypoxic conditions has been reported to play an anti-infiltratory role in GBM [47]. Furthermore, the overexpression of an *NPAS2* variant was associated with poor patient outcomes in GBM [43].

Despite the above described knowledge, a comprehensive analysis in terms of circadian regulation during glioma progression is still missing. Here we aim to characterize the dysregulation of the circadian clock and clock-regulated genes in a glioma context. We analysed expression patterns of differentially expressed clock-regulated genes in LGG and GBM, further reconstructed progression patterns of mutations in LGG and GBM using single-sample data, and additionally the evolution of LGG in individual patients using multi-sample data. Our results show a higher number of differentially expressed clock-regulated genes in GBM as compared to LGG, highlighting the higher level of dysregulation in GBM. We further show that exploring the expression of 45 differentially expressed clock-regulated genes in GBM suffices to distinguish tumor from normal tissue. In particular, three of the 45 differentially expressed clock-regulated genes in GBM belong to the core-clock network, namely *RORB*, *NR1D1* and *CRY2*, which are all downregulated in GBM. When focusing on clock regulated drivers in glioma, our results further reveal that in LGG, an early small-scale mutation (SSM) in *TP53* is followed by either the loss of *TERT* or the loss of *APC*. The loss of *HIF1A* is an early event, while the loss of *TP53* and SSM in *APC* occur late in the LGG progression. On the contrary, in GBM, the loss of *TP53* is an earlier event, and the losses of *TERT* and *APC* occur later. In the multi-sample LGG dataset, we observe whole-genome doubling (WGD) events exclusively subclonally, as well as frequent subclonal gains and losses of *APC*, *HIF1A*, *TERT* and *TP53.* This might indicate the role of the four clock-regulated driver genes in the subclonal diversification and intra-tumour heterogeneity of LGG.

Our results highlight the relevance of circadian regulation in gliomas and the need to consider circadian knowledge in future clinical studies for brain tumours.

## Methods

### Data source for RNA-seq data sets

RNA-seq expression data for low-grade glioma (LGG) and glioblastoma multiforme (GBM) were obtained from TCGA (https://portal.gdc.cancer.gov/), accessed on 27.03.2022) using the R package *TCGAbiolinks* [48, 49]. The legacy version of RNA-seq aligned to the hg19 was retrieved, selecting for data category *Gene expression*, data type *Gene expression quantification*, experimental strategy *RNA-seq*, file type *results*, and platform *Illumina HiSeq.* The GBM dataset consists of 169 primary tumour samples, and the LGG dataset consists of 534 primary tumour samples of grade 2 (n = 216), grade 3 (n = 241) and unknown grade (n = 77). 5 normal samples were provided as part of the GBM dataset. LGG dataset contains 241 samples from females, 292 samples from males and one sample from an unknown sex. GBM dataset contains 59 samples from females, 109 samples from males and 6 samples from unknown sex. Age range of patients in LGG dataset is between 14 and 87 years old, and in the GBM dataset between 21 and 89 years old.

### Data source for mutation data sets

Legacy data with simple somatic mutations (SSMs) were obtained using the *TCGAbiolinks* [48, 49] R package and selecting TCGA-GBM and TCGA-LGG projects, *Simple nucleotide variation* as data category, *Simple somatic mutation* as data type, open access, and file types ‘ucsc.edu_GBM.IlluminaGA_DNASeq_automated.Level_2.1.1.0.somatic.maf’ for TCGA-GBM and “LGG_pairs.aggregated.capture.tcga.uuid.automated.somatic.maf” for TCGA-LGG. Legacy copy number alterations were obtained using the function *getFirehoseData* from the *TCGAbiolinks* [50]. R package by selecting the GBM and LGG datasets, GISTIC and choosing the last available analysis date ‘20160128’. LGG dataset contains 230 samples from females, 285 samples from males and one sample from an unknown sex. GBM dataset contains 229 samples from females, 354 samples from males and 4 unknown samples. Age range of patients in LGG dataset is between 14 and 87 years old, and in the GBM dataset between 10 and 89 years old.

### Data source for multi-sample datasets (JPN-LGG)

Previously published Japanese LGG (JPN-LGG) data set [50] was obtained from the European Genome-phenome Archive (EGA), accession code EGAS00001001044. The data set consists of 14 patients with grade 2 and 3 gliomas from whom multiple samples were taken. For 4 patients, 5–9 spatially separated samples were collected during a single surgery, while for 10 patients, 2–4 samples were taken across different surgeries, spanning a maximum of 61.8 months after the initial surgery. Of 10 patients for which time separated samples were available, 1 was female and 9 were male.

### Differential gene expression

The *limma* package [51] was used to derive a set of differentially expressed genes in both LGG and GBM data sets. After the raw counts were retrieved, tumour samples with unknown grade were removed. Low expressed genes were filtered out using the *edgeR* [52] R package and Trimmed Mean of M-values (TMM) normalization was performed on raw counts using the same package. After applying voom to normalized counts, we obtained two sets of differentially expressed genes by comparing 169 GBM tumours and 457 LGG tumours with 5 normal samples, applying cut-offs *p* < 0.05, *q* < 0.05 and selecting an absolute log_2_-fold change greater than 1. Since we are limited to 5 normal TCGA samples, we additionally obtained a list of differentially expressed genes in TCGA-LGG and TCGA-GBM using GEPIA2, which compares tumour data from TCGA-LGG and TCGA-GBM to 207 normal brain samples from GTEx [49]. In GEPIA2 [53] we selected the *limma* approach with the |logFC| > 1 and the *q* < 0.05. Finally, we intersected the set of genes obtained with *limma* [51] and GEPIA2 [53] and selected clock-controlled genes of interest. The set of 185 genes of interest was constructed by combining the network of clock-regulated genes (NCRG) [41], and clock-controlled genes obtained from Sulli et al. [54]. The list of 185 genes of interest is provided in Additional file 1.

The heatmap of differentially expressed genes of interest was generated based on z-scores, as follows: $z=\frac{mean\left(tumor\right)-mean\left(normal\right)}{sd\left(data\right)}$. The clustering of rows (genes) and columns (samples) was performed based on the Pearson correlation. The gene set and pathway enrichment plots were created using the R package clusterProfiler.

### Survival analysis

Univariate Cox regression analysis was performed to explore the effect of sex, tumour grade and IDH mutation status on overall survival. We selected patients in TCGA-LGG and TCGA-GBM for which vital status, days until death or days until last follow up, sex, IDH mutation status and tumour grade were known. We obtained a dataset of 306 patients, with 233 patients with wild-type IDH, 73 patients with mutant IDH, 138 patients with grade 2 glioma, 146 patients with grade 3 glioma and 22 patients with grade 4 glioma. We found that sex does not influence overall survival (*p* = 0.706), while tumour grade (*p* = 7⨯10^− 15^) and IDH mutation status (*p* ≤ 2⨯10^− 16^) effect overall survival, with patients with higher grade tumours and wild-type IDH having worse prognosis. Further, the set of differentially expressed genes of interest in LGG and GBM, and additionally genes belonging to the core-clock network were analyzed for their effect on survival. Cox proportional hazards regression model was fitted to the expression data, stratified based on separately tumour grade and IDH mutation status, and survival assessed using clinical variables describing the last follow-up and days until death. The analysis was performed using the *survival* R package [55]. The expression of each gene in tumour samples was classified as either high or low, based on the median expression. 17 genes were significantly associated with patient overall survival (*q* < 0.05) when stratifying for tumour grade, and 12 when stratifying for IDH mutation status.

### Gene set and pathway enrichment analysis

Gene set enrichment (GSEA) and pathway enrichment analysis were performed using R package *clusterProfiler* [56, 57]. GSEA was performed for biological process, molecular function, and cellular component ontologies, and performing Benjamini-Hochberg p-value adjustment method and selecting *q* < 0.05.

### Progression pattern reconstruction

CAPRI [58] was used to reconstruct progression patterns from individual samples in TCGA- LGG and TCGA-GBM. As input for CAPRI [58], we selected a set of cancer drivers associated with glioma from COSMIC [59], that occur in at least 5% samples.

### Copy number calling and phylogenetic tree reconstruction

The whole exome sequencing data from the JPN-LGG dataset were pre-processed using *samtools* [60] by removing unmapped and duplicated reads and filtering out reads with mapping quality < 20. Copy numbers were called using *Sequenza* [61]. Samples with an estimated tumour cell composition < 20% were discarded. Furthermore, the quality control of copy number calls was estimated by manual inspection. As a result, phylogenetic trees of 13 individual tumours, based on a total 55 samples, were reconstructed using MEDICC2 [62].

## Results

### GBM shows higher level of dysregulation in gene expression then LGG as compared to control

**Fig. 1:**
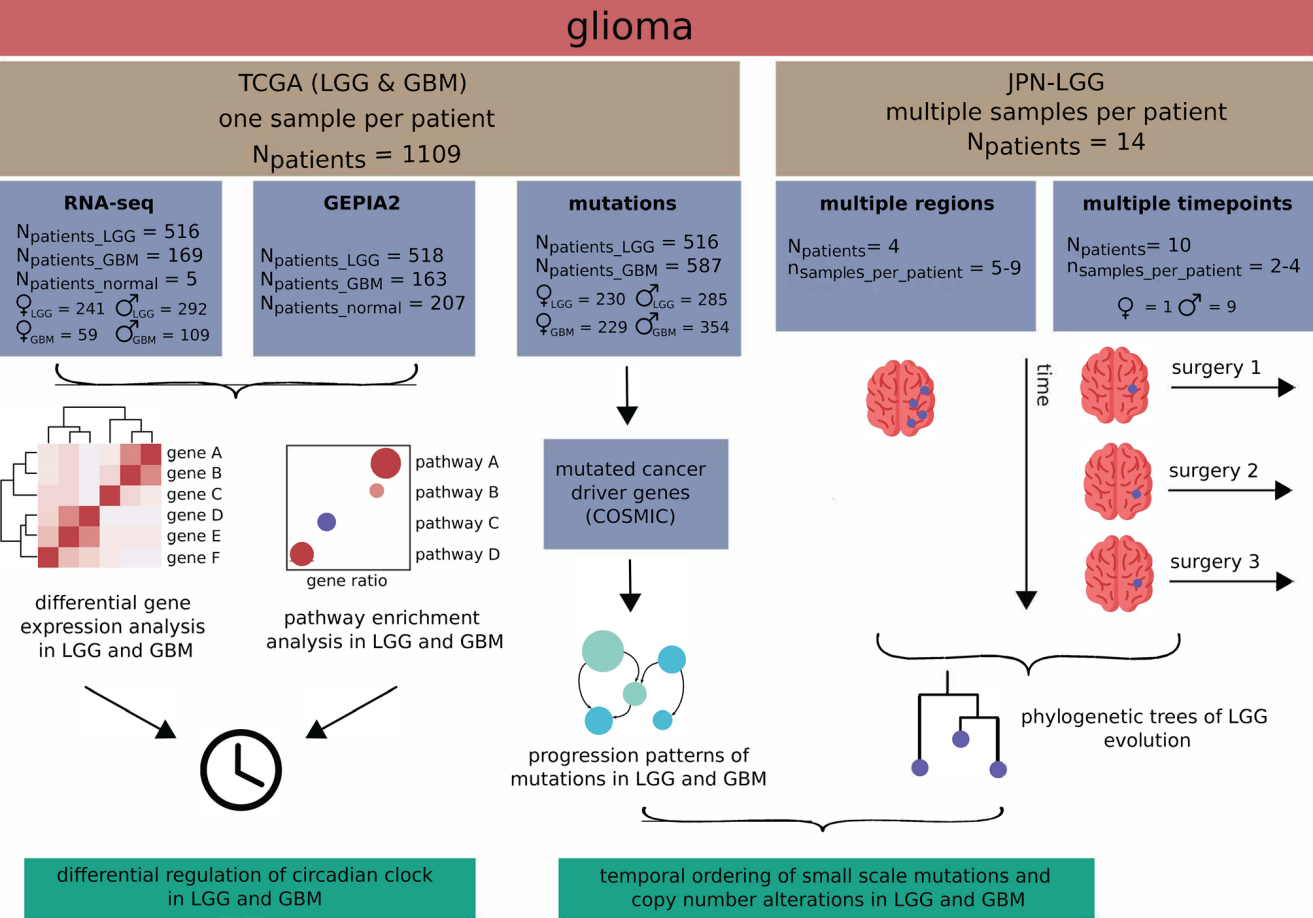
Analysis pipeline of single sample data from TCGA-LGG and TCGA-GBM, and a multi-sample JPN-LGG dataset. A read count matrix obtained from RNA-sequencing experiments for LGG (631 patients) and GBM (169 patients) was used to generate a list of differentially expressed genes (tumour vs. normal samples). The resulting list was intersected with the list of differentially expressed genes retrieved from GEPIA2 [17]. The final list of differentially expressed genes in LGG and GBM was analysed for enriched KEGG pathways, and the analysis of expression patterns of genes of interest. A list of genes affected by SSMs or CNAs in LGG and GBM was obtained. From the list, driver genes with annotated mutations, losses or gains were selected and used for the reconstruction of progression patterns in LGG and GBM. Phylogenetic trees were reconstructed based on the mutations and CNAs from multi-sample data in JPN-LGG cohort. The temporal ordering of alterations in genes of interest was obtained from the phylogenetic trees in JPN-LGG and progression patterns in the TCGA-LGG and TCGA-GBM

We explored the existence of differential expression regulation on LGG and GBM, compared to normal samples, based on the expression of 185 clock-regulated genes of interest. The set of genes of interest consists of a curated list of genes, which includes the genes contained in a previously assembled network of clock-regulated genes (NCRG) [41], combined with an updated list of clock-controlled genes in cancer reported by Sulli and colleagues [54]. We investigated the alterations on the expression of these genes in RNA-sequencing data from TCGA-LGG and TCGA-GBM data sets (Fig. 1).

**Fig. 2:**
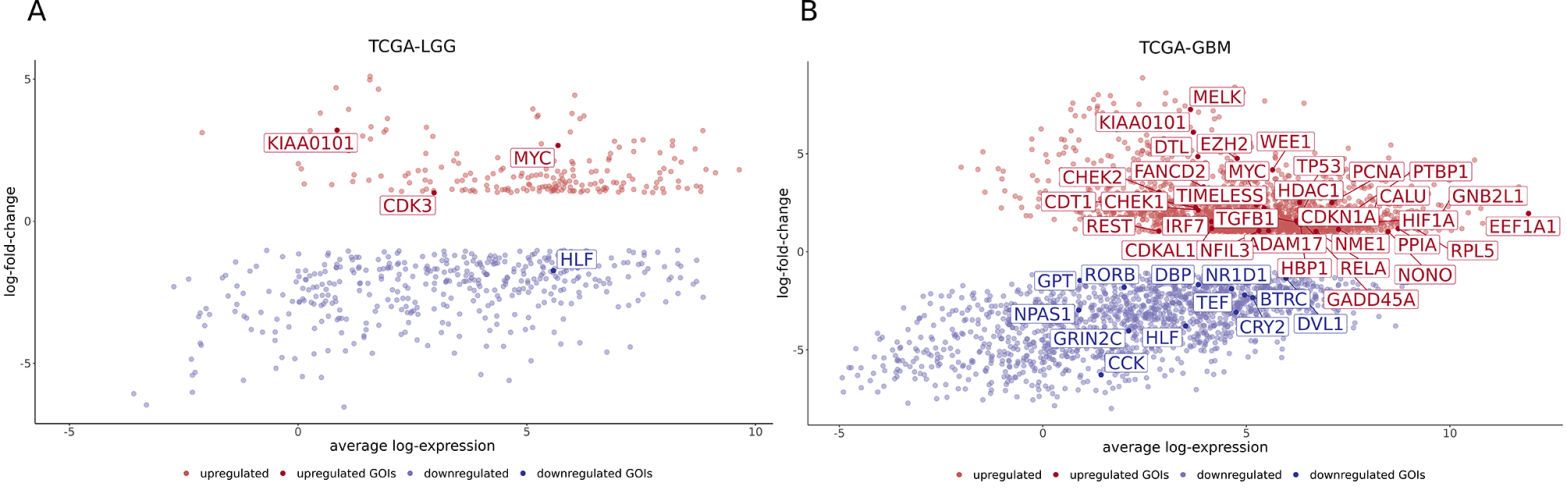
Common differentially expressed genes in TCGA-LGG and TCGA-GBM datasets. (**A**) Mean-difference plot of 610 differentially expressed genes in TCGA-LGG dataset. Depicted are significantly upregulated genes (logFC > 1, q < 0.05), in red, and significantly downregulated genes (logFC < -1, q < 0.05), in blue. Significantly differentially expressed genes of interest (|logFC| > 1, q < 0.05) are annotated (four genes). (**B**) Mean-difference plot of 3519 differentially expressed genes in TCGA-GBM dataset. Depicted are significantly upregulated genes (logFC > 1, q < 0.05), in red, and significantly downregulated genes (logFC < -1, q < 0.05), in blue. Significantly differentially expressed genes of interest (|logFC| > 1, q < 0.05) are annotated (45 genes)

We first analysed the degree of similarity of normal samples compared to LGG and GBM datasets by performing unsupervised clustering of patient samples in TCGA-LGG and TCGA-GBM based on the expression of all genes (Supplementary Fig. 1). While normal samples and LGG samples seem to have a higher degree of similarity, pointing to less alterations in LGG compared to the normal samples, GBM samples show a separation from normal samples into two distinct clusters. We further extracted differentially expressed genes (tumour vs. normal samples) in TCGA-LGG (Fig. 2A) and TCGA-GBM (Fig. 2B). The set of differentially expressed genes was obtained by intersecting the differentially expressed genes from the comparison with 5 normal samples from TCGA and the differentially expressed genes obtained using GEPIA2, which compares tumour data from TCGA to 207 normal samples from human brain, deposited in GTEx [53] (Fig. 1). Our results show that a higher number of genes are differentially expressed in GBM (3519 genes) compared to control datasets, as in LGG (610 genes) (Fig. 2). In LGG, four genes of interest are differentially expressed (Fig. 2A), of which *KIAA0101*, *CDK3*, *MYC* are upregulated and *HLF* is downregulated. *KIAA0101* is one of the regulators of cell invasion [63, 64], *CDK3* is involved in cell cycle [65], *MYC* is involved in cell growth and proliferation, differentiation, and programmed cell death [66]. *HLF* regulates the development of hematopoietic cells and malignant transmission and is involved in the resistance to cell death [67]. In GBM, 45 genes of interest are differentially expressed (Fig. 2B), of which 12 are downregulated and 33 upregulated. The downregulated genes are part of the core-clock network (*RORB*, *NR1D1*, *CRY2*), involved in malignant transmission and resistance to cell death (*HLF*), modulation of cell cycle (*BTRC*), signal transmission in nervous system (*CCK*), metabolism of glucose and amino acids (*GPT*), glutamatergic signalling (*GRIN2C*) and Wnt signalling (*DVL1*). Two genes (*DBP*, *TEF*) are paralogs of *HLF*, which is also downregulated in LGG. Upregulated genes regulate cell cycle (*MELK*, *DTL*, *EZH2*, *WEE1*, *CHEK1/2*, *PCNA*, *CDKN1A*), maintain cellular homeostasis (*TP53*, *TIMELESS*), DNA damage repair and negative ferroptosis regulation (*FANCD2*), cell growth and proliferation, differentiation and programmed cell death (*MYC*), regulation of gene transcription (*HDAC1*, *REST*, *IRF7*, *NFIL3*, *HBP1*, *NONO*), regulation of neural precursor cell differentiation (*PTBP1*) and alternative splicing (*PTBP1*, *NONO*), regulation of tissue homeostasis and glioma stem cell regulation (*TGFB1*), endoplasmic reticulum-related functions such as protein folding and sorting (*CALU*), recruitment, assembly and/or regulation of a variety of signalling molecules (*GNB2L1*), DNA replication and mitosis (*CDT1*), cellular response to hypoxia (*HIF1A*), delivery of aminoacyl tRNAs to the ribosome (*EEF1A1*), unknown function (*CDKAL1*), metastasis suppression (*NME1*), ribosome construction and tumorigenesis regulation (*RPL5*), neural progenitor cell migration and neurogenesis (*ADAM17*), response to the stressful growth arrest and response to the treatment with DNA-damaging agents (*GADD45A*), protein folding (*PPIA*), regulation of cell survival, immune and inflammatory responses (*RELA*). Only one gene of interest is LGG-specific – *CDK3*.

Interestingly the core-clock genes *RORB*, *NR1D1*, and *CRY2* are among the set of downregulated genes in GBM. Our results indicate that, based on the differentially expressed genes of interest, GBM shows a higher level of dysregulation compared to LGG.

### Differentially expressed genes of interest separate tumour and normal tissue in GBM patients

**Fig. 3:**
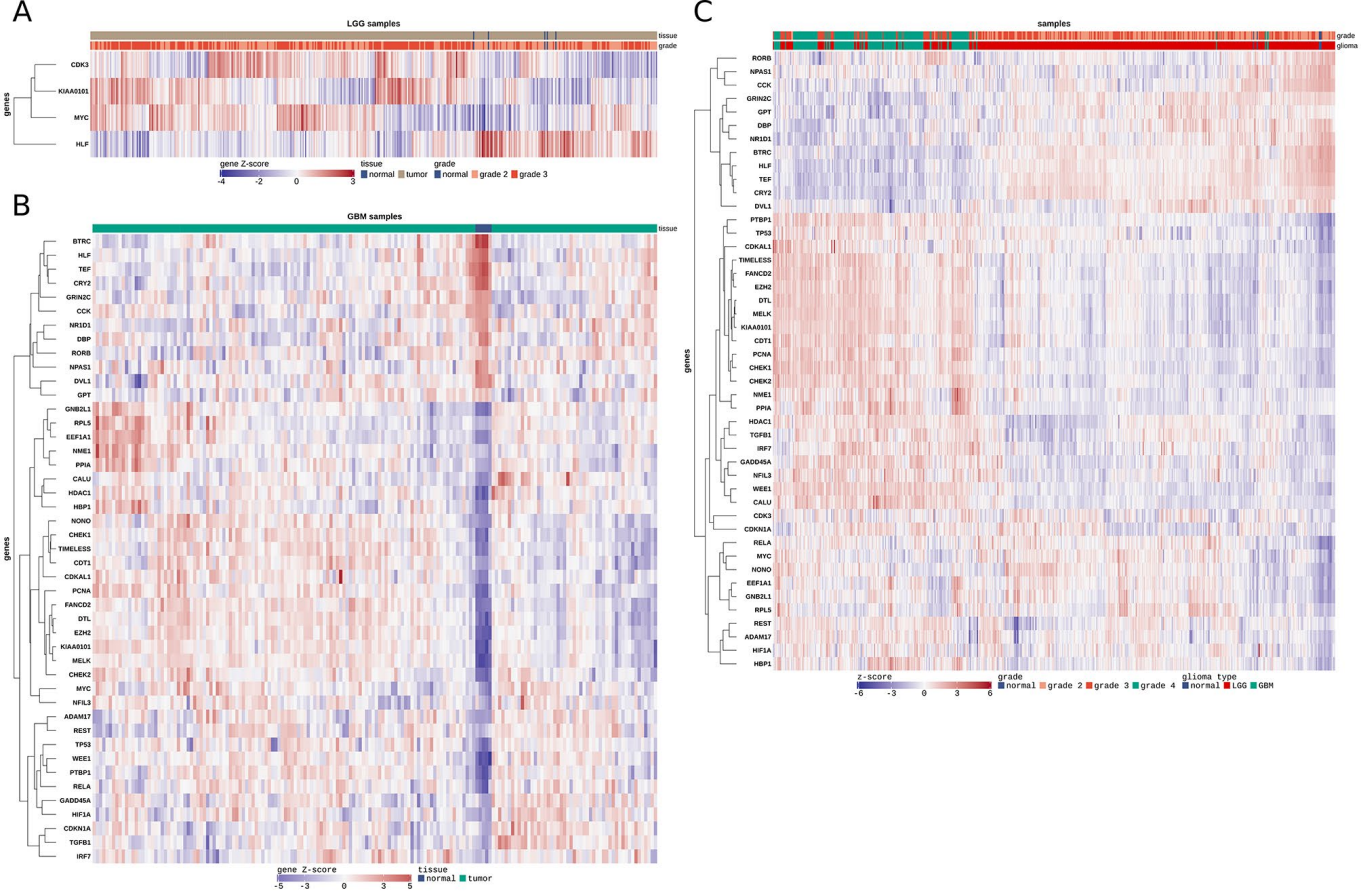
Hierarchical clustering of differentially expressed genes of interest in TCGA-LGG and TCGA-GBM. (**A**) Hierarchical clustering of 4 differentially expressed genes of interest (|logFC| > 1, *q* < 0.05). Normal samples are marked in blue and tumour samples in brown. Orange indicates grade 2, and red grade 3 glioma. (**B**) Hierarchical clustering of 45 significantly differentially expressed genes of interest (|logFC| > 1, *q* < 0.05). Normal samples are marked in blue and tumour samples in brown. (**C**) Hierarchical clustering of 46 differentially expressed genes of interest in LGG and GBM. Normal samples are marked in blue, grade II in orange, grade III in red and grade IV in green

To further investigate the differentially expressed genes of interest across different samples in LGG and GBM, we performed hierarchical clustering on the expression of the 46 genes of interest (Fig. 3**).** Out of that subgroup, 45 differentially expressed genes of interest in GBM allowed to distinguish tumour from normal samples (Fig. 3B). These genes group into 2 clusters that contain 12 and 33 genes based on their expression in normal samples. 4 differentially expressed genes of interest in LGG (*KIAA0101*, *CDK3*, *MYC* and *HLF*) do not separate tumour from normal samples (Fig. 3A). However, hierarchical clustering of all 46 differentially expressed genes of interest in both LGG and GBM reveals a tendency to separate lower-grade gliomas from the higher-grade tumours (Fig. 3C). Most grade IV gliomas (GBM) are found on the left side of the heatmap together with grade III gliomas (LGG). Grade II gliomas (LGG) are predominantly found on the right side of the heatmap, together with normal samples and grade III gliomas (LGG). 3 genes of interest (*HLF*, *MYC* and *KIAA0101*) are differentially expressed both in LGG and GBM. *KIAA0101* is upregulated in most samples in higher grade gliomas and downregulated in lower grade gliomas. *HLF* shows the opposite tendency: lower expression in higher-grade gliomas and higher expression in lower-grade gliomas. *MYC* does not show altered expression patterns in correlation to tumour grade.

We further analysed the expression patterns of the top 100 differentially expressed genes in LGG (Supplementary Fig. 2A) and GBM (Supplementary Fig. 3B), compared to normal tissue. Unlike in GBM, it is not possible to distinguish tumour from normal samples using the top 100 differentially expressed genes in LGG (Supplementary Fig. 2A).

We also examined gene sets and pathways enriched in LGG (Supplementary Fig. 2B-2 C) and GBM (Supplementary Fig. 3B-3E) data sets, by conducting a gene set enrichment analysis (GSEA) based on all differentially expressed genes. Biological processes enriched both in LGG and GBM contain upregulated cell cycle-related terms (LGG: regulation of cell cycle, mitotic cell cycle process and cell cycle; GBM: mitotic cell cycle, G1/S and G1/M transition of mitotic cell cycle), as well as terms related to cellular process (LGG: cell division; GBM: DNA repair, DNA replication, DNA metabolic process, cell activation, nuclear division, microtubule cytoskeleton organization). LGG specifically shows upregulation of RNA metabolic process and chromosome organization, and GBM data sets show upregulation of immune effector process. Moreover, GBM shows downregulation of cell communication (chemical synaptic transmission, cell-cell signalling, G protein-coupled receptor signalling pathway), system process, cation transport, behaviour, and neuron differentiation processes. In addition, the G protein-coupled receptor signalling pathway is upregulated in LGG. For GBM, 6 terms related to chromosome (chromosome and chromosomal region), intracellular anatomical structure (secretory and cytoplasmic vesicle lumen, nuclear protein-containing complex) and vesicle are upregulated. 9 downregulated terms in GBM are related to membrane (ion, and cation channel complex, integral and intrinsic component of plasma), somatodendritic compartment (dendrite, neuronal cell body) and neuron projection (axon). We found 12 terms related to molecular function that contain genes of interest in their core enrichment for GBM (Supplementary Fig. 3C), of which three terms, related to binding and nucleic acid binding, are upregulated (RNA, DNA, and chromatin binding). 9 terms related to transmembrane transporter activity (voltage-gate ion, cation, and ion channel activity, channel activity, inorganic molecular entity, cation, and ion transmembrane transporter activity, transported activity, and transmembrane signalling receptor activity) are downregulated. The KEGG pathway enrichment analysis resulted in 28 enriched pathways, from which the top 10 most significantly enriched pathways, include the p53 signalling pathway, cell cycle, cellular senescence, Epstein-Virus infection, human T-cell leukaemia virus 1 infection and herpes simplex virus 1 infection, and contain genes of interest in their core enrichment (Supplementary Fig. 3E), all downregulated. We also compared the expression of *NR1D1, CRY2, RORB, DBP, TEF, MYC, WEE1, BMAL1, CLOCK*, and *PER1-3* in LGG and GBM samples, as shown in the Supplementary Fig. 4.

In summary, exploring the expression of 45 genes of interest allows to distinguish GBM from non-cancer tissue. Not surprisingly, following the results in Fig. 2 that show a comparably high number of differentially expressed genes in GBM, the GSEA analysis also reveals a higher number of enriched terms typically associated with cancer in GBM compared to LGG. These results are further reinforced by the KEGG pathways analysis, which showed enriched pathways for differentially expressed genes only in GBM.

**Fig. 4:**
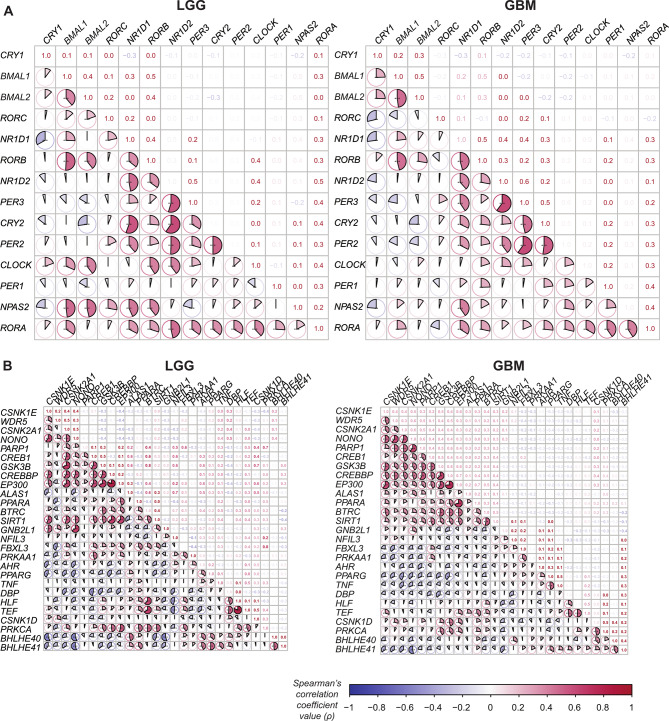
Correlation heatmaps of CCN and ECCN genes in LGG and GBM samples. (**A**) Heatmap representing co-expression patterns for LGG (left side) and GBM samples (right side) for CCN genes. (**B**) Heatmap representing co-expression patterns for LGG (left side) and GBM samples (right side) for ECCN genes. In A) and B), colour and sizes of the pie charts indicate the strength and the sign of correlation; *p < 0.05, ** p < 0.01, ***p < 0.001

Next, we explored the co-expression patterns of genes from the core-clock network (CCN) and a published network known to be regulated directly by the core-clock, the extended core clock network (ECCN) [41], and our cancer-associated genes of interest in both glioma subtypes. For this purpose, we performed a Spearman correlation analysis of these short-listed genes in LGG and GBM samples. To prevent the redundancy due to overlapping genes between CCN and other clock-controlled genes of interest, the genes overlapping with the CCN list (Fig. 4A) were not plotted again as part of other genes of interest (Fig. 4B and Additional file 1). Our results show a loss of correlation strength regarding elements of the circadian clock network in GBM compared to LGG. We also observed that in LGG more core-clock genes were significantly correlated whereas in GBM these significant correlations were lost pointing to a stronger disruption of the core-clock network in GBM (Fig. 4A**).** Notably, our results suggested that the co-expression of PER family genes (*PER1*, *PER2* and *PER3*) showed the most drastic changes, within the CCN, between LGG and GBM. These included a loss of significant correlation for *PER2-RORC*, and resulted in opposite correlation patterns for *PER1-CLOCK*, *PER3-RORC*, *PER1-BMAL2* between LGG and GBM. Furthermore, 34 ECCN gene pairs show a switch in their correlation type, between LGG and GBM.

**Fig. 5:**
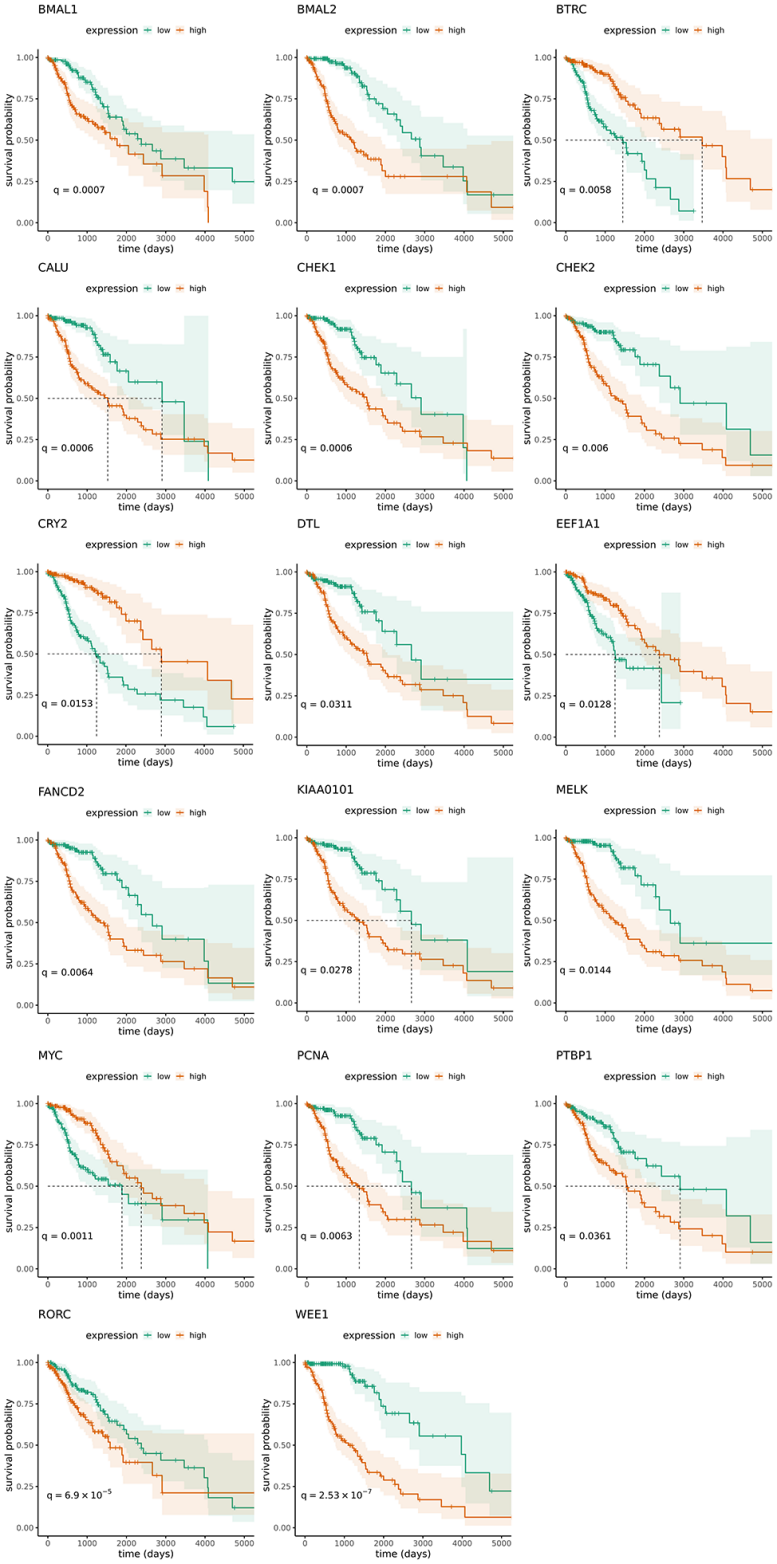
Survival analysis of differentially expressed genes of interest and CCN genes. Kaplan-Meier curves for 17 genes with a significant effect on survival (q < 0.05). Green shows low and red high expression

We further evaluated the impact of the 46 differentially expressed genes in LGG and GBM, as well as CCN genes on overall survival for glioma patients. In particular, we investigated the effect of sex, tumour grade and IDH mutation status on overall survival. Our results indicate that sex does not show an effect on overall survival (*p* = 0.706), while higher tumour grade (*p* = 7⨯10^− 15^) and IDH mutation status vs. wild-type IDH (*p* ≤ 2⨯10^− 16^) had a significant impact on overall survival. Further, a Cox regression model with stratification based on tumour grade was carried out, and 17 genes were found to have a significant effect on survival (*q* < 0.05) (Fig. 5). Strikingly, four of these genes are core-clock genes, namely *BMAL1/2, CRY2* and *RORC*. The remaining 40 genes, which did not show a significant effect on survival are depicted in the Supplementary Fig. 5.

Low expression of 13 genes is significantly correlated with better survival. These include the CCN genes (*BMAL1/2*, *RORC*), and *WEE1*, *PTBP1*, *PCNA*, *KIAA0101*, *FANCD2*, *DTL*, *MELK*, *CHEK1/2* and *CALU*. Higher expression of *MYC*, *BTRC*, *EEF1A1* and *CRY2* significantly correlates with better survival.

We further fitted Cox regression analysis while stratifying for IDH mutation status, and found 12 genes that have an effect on overall survival (Supplementary Fig. 6).

### Tumour suppressor APC and a catalytic subunit of telomerase, TERT, are lost late in the progression of GBM

**Fig. 6:**
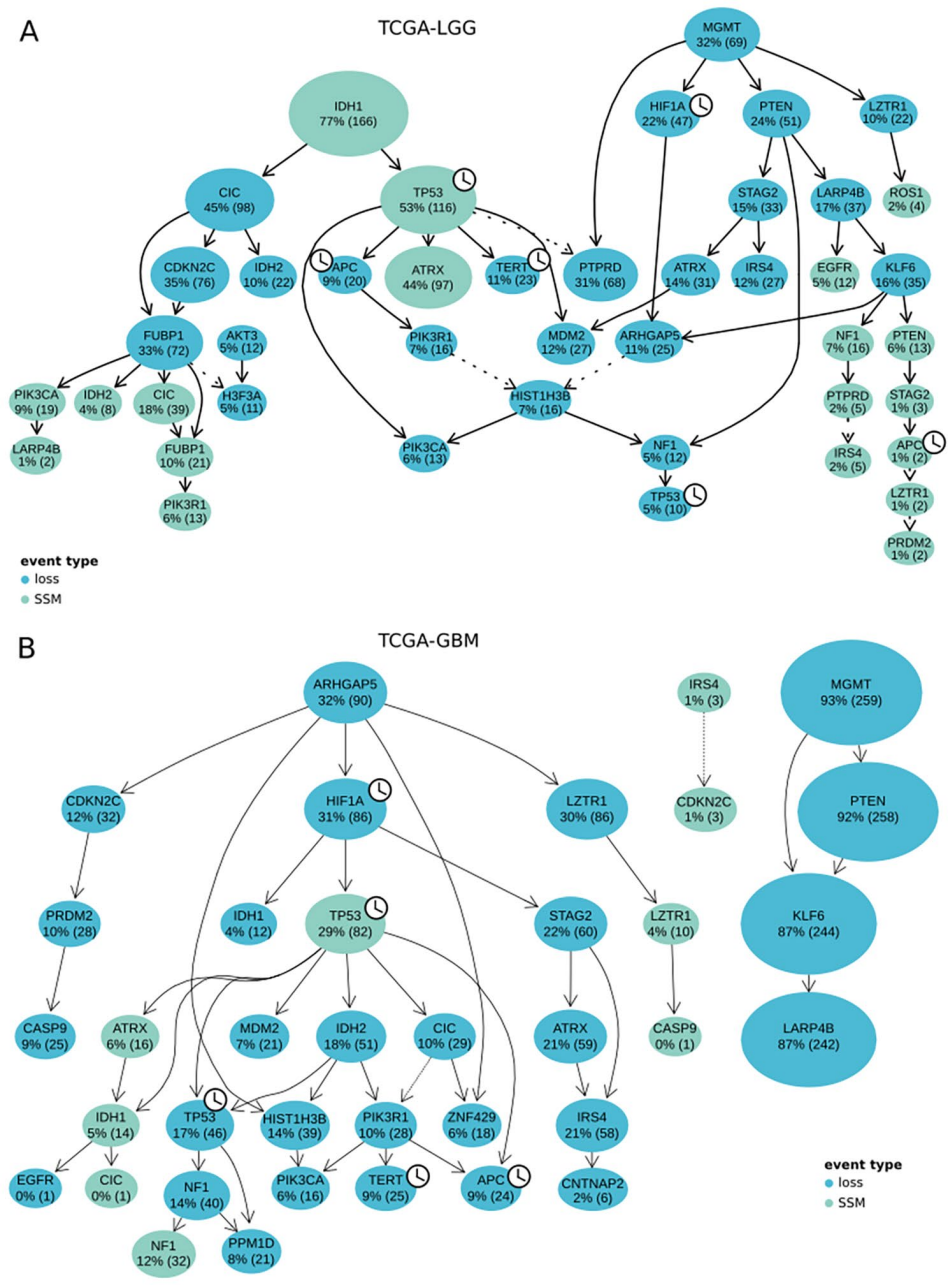
Progression patterns in mutated COSMIC genes in LGG and GBM. (**A**) Progression patterns in mutated COSMIC genes in LGG. (**B**) Progression patterns in mutated COSMIC genes in GBM. In (**A**) and (**B**), losses are shown in blue and mutation events (SSMs) in green. The percentage in each circle indicates the proportion of samples in which the event occurred, with the associated number of samples in parentheses. The size of the node indicates the relative frequency of the event in the dataset. The clock icon indicates a COSMIC gene which is part of the genes of interest. Dashed lines indicate edges for which one of three edge confidence measures had a p-value > 0.01. In (**A**), the hypergeometric test p-value for TP53 and PTPRD edge is 0.06. Temporal priority between MGMT and PTPRD is 0.08, between PTPRD and IRS4 0.31, between AKT3 and H3F3A 0.13, between LARP4B and LZTR1 0.46 and between LZTR1 and APC 0.46. In temporal priority between CDKN2C and IRS4 is 0.38

To further investigate the alterations in the genes of interest during the progression of LGG and GBM, we employed the dataset with annotated mutations from TCGA-LGG and TCGA-GBM (Fig. 1). Events (small scale mutations (SSMs), losses and gains) that occur in at least 5% of samples were filtered out, resulting in the set of SSMs and losses in both TCGA-LGG and TCGA-GBM datasets. Before filtering, gain of *PPM1D* in one patient was present in LGG. We selected genes with driver mutations in gliomas from COSMIC [59] and reconstructed the mutation patterns for these genes during glioma progression using CAPRI [58] (Fig. 6). The reconstructed evolutionary patterns contain 4 genes of interest *TP53*, *APC*, *TERT* and *HIF1A*. Of these, *TP53* and *HIF1A* are differentially expressed in GBM (Fig. 3B).

The reconstructed progression pattern in LGG showed three origins **(**Fig. 6A**)**. Of 6 possible events in the genes of interest, 3 of them occur in the first steps of tumour progression. According to our results, the first alteration of the LGG progression pattern is the mutation in *IDH1* present in 76% of samples. Mutations in *IDH1/2* are characteristic for lower-grade gliomas and are associated with a better prognosis. The mutation of *IDH1* is further followed by the SSM in *TP53*, and further by the loss of *APC* and *TERT*. This event is followed by a loss of *MGMT*, present in 32% of samples, followed by the loss of *H1F1A* in 21% of samples. The third pattern consists only of two events, starting with the loss of *AKT3* in 5% of samples, followed by the loss of *H3F3A* in 5% of samples. For GBM, our results point to three reconstructed progression patterns **(**Fig. 6B**)**. The largest one consists of 31 nodes and contains all events in genes of interest. It starts with the loss of the *ARHGAP5* in 32% of samples, followed by the loss of *HIF1A* in 31% of the samples, and the SSM in *TP53* in 29% of the samples. The pattern also exhibits losses of *TP53*, *TERT*, and *APC*, but later in the evolution pattern.

To complement our analysis, we explored the time-dependent expression of the four glioma driver genes (*APC*, *HIF1A*, *TERT* and *TP53*) in HCT116 wild type and HCT116 KO cell lines for the core-clock genes *BMAL1*, *PER2* and *NR1D1*, as well as their differential expression in each of the KOs compared to the WT cells (Supplementary Fig. 7). *APC *is downregulated in all clock KOs, while TP53 is downregulated in the *BMAL1* and *PER2 *KO cells and upregulated in the *NR1D1* KO. *HIF1A* and *TERT* are upregulated in the KO cells. In agreement with our findings, suggesting a clock regulation of these genes, our results for the HCT116 cells showed indeed a time-dependent expression of *APC*, *HIF1A*, *TERT* and *TP53* in each of the KOs. Moreover, these genes are also differentially expressed between each KO condition and the WT cells, reinforcing our finding regarding the potential clock-regulated expression of these genes, albeit in a different cell type.

Comparing the reconstructed progression patterns of mutations in COSMIC driver genes in LGG and GBM, it is evident that *APC* and *TERT* losses occur later in GBM. Moreover, *APC* does not seem to harbour SSMs in GBM, as opposed to our observations for LGG.

### Genes of interest exhibit frequent subclonal events in LGG evolution

**Fig. 7:**
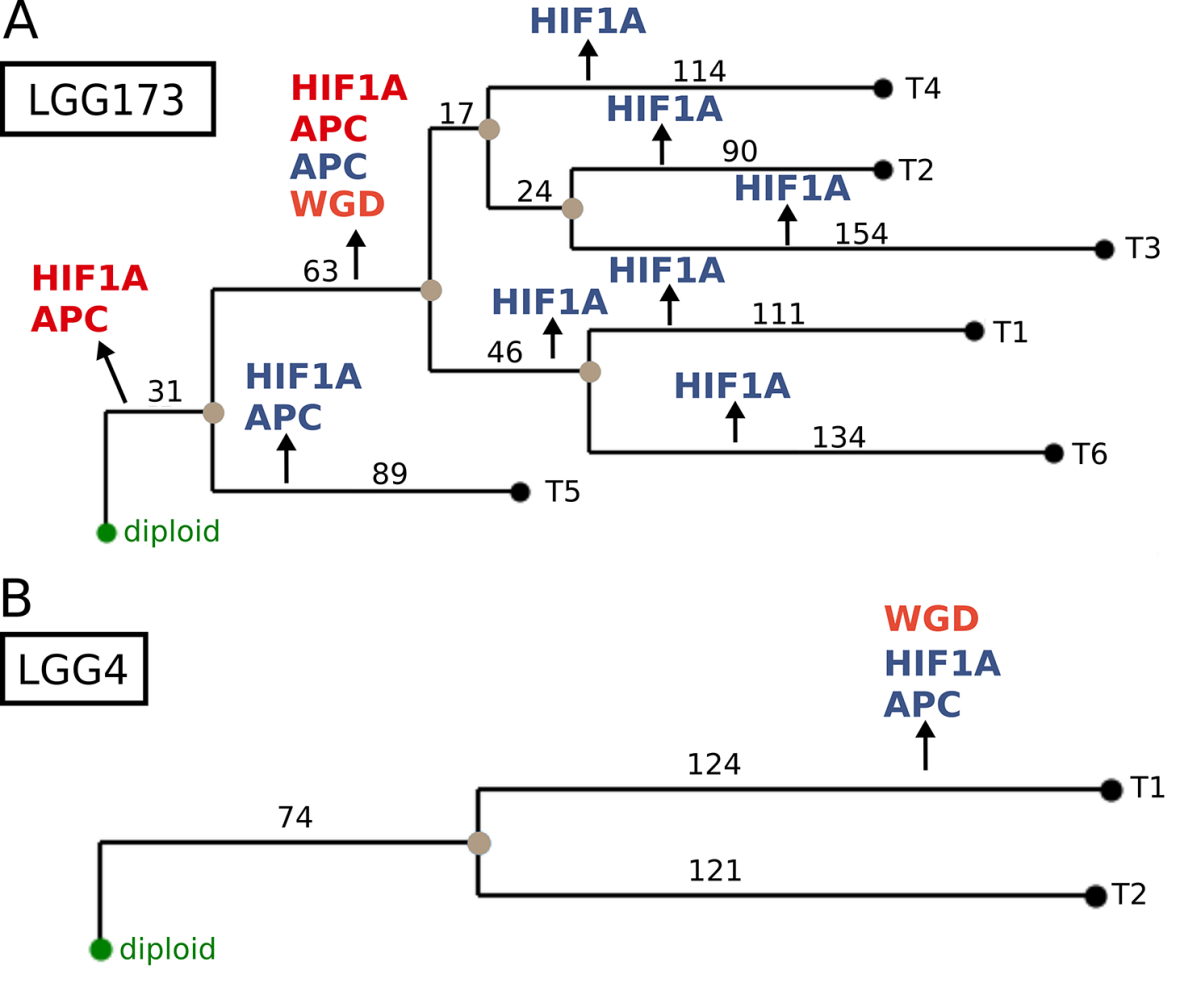
Evolutionary trees for two JPN-LGG patients. Evolutionary tree for (**A**) a patient LGG173 with 6 samples taken at the same surgery and (**B**) a patient LGG4 with 2 samples taken over 2 surgeries. Diploid (normal) node is marked in green, internal nodes in brown and leaves in black. Genes with gains are indicated in red and with losses in blue. WGD is indicated in orange. Numbers on branches indicate the distance of adjacent nodes in terms of number of events of gains and losses

Exploring evolutionary patterns of tumours using only a single sample per patient has several limitations [68], to overcome this problem, we investigated the role and position of the driver genes of interest in the evolution of LGG, using a multi-sample JPN-LGG dataset (Fig. 1). We reconstructed the evolution of 13 individual LGGs using multiple samples retrieved from the same patient. Our results highlight the clonality of events affecting 4 genes of interest (*TERT*, *TP53*, *APC* and *HIF1A*) from the set of driver genes in gliomas [59]. Figure 7 shows LGG evolutionary trees for two patients - patient LGG173 from whom 6 samples were retrieved during the same surgery (Fig. 7A) and patient LGG4, from whom two samples were retrieved during two different surgeries (Fig. 7B). The reconstructed evolutionary trees for 11 patients are provided in Supplementary Fig. 8. Patient LGG173 exhibits a clonal gain of *HIF1A* and *APC*, followed by the subclonal whole-genome doubling (WGD) event and another subclonal gain of *HIF1A* and *APC*, as well as the subclonal loss of *APC*. The subclonal loss of *APC* is followed by the subclonal loss of *HIF1A* in five out of six samples (Fig. 7A). Patient LGG4 shows a subclonal WGD event and a subclonal loss of *HIF1A* and *APC* on the same branch.

Comparing the evolutionary trees of the 13 patients in JPN-LGG dataset, we observed a clonal gain of *APC* in 2 patients and its clonal loss in 1 patient, as well as a clonal gain of *HIF1A* in 3 patients and its clonal loss in 1 patient. *TP53* experienced a clonal loss in 1 patient. Interestingly, genes of interest seem to be more frequently affected by subclonal events, especially in the case of *APC* and *HIF1A*. *TERT* is subclonally gained in 2 patients and lost in 2 patients, while *TP53* is subclonally lost in 2 patients and subclonally gained in 1 patient. *APC* shows subclonal losses in 6 patients and gains in 7 patients. In three of these patients, *APC* is both lost and gained subclonally. *HIF1A* exhibits subclonal gains in 8 patients and losses in 5 patients. Of these, 5 patients harbour both subclonal gains and losses of *HIF1A*. Moreover, the *WGD* event occurs in 7 patients and is exclusively subclonal.

Taken together, the abundance of subclonal events affecting genes of interest in LGG is evident. This might indicate a strong subclonal diversification of individual tumours and reflect high intra-tumor heterogeneity previously described in gliomas.

## Discussion

Glioblastomas (WHO grade IV) are the most frequent malignant brain tumours [69]. Due to their distinct pathophysiological features cure is impossible, even with the use of multimodal therapies [17–19]. Despite recent advances in defining molecular characteristics of these tumours, targeted therapies are far from clinical application. So far circadian rhythms have been shown to be dysregulated at the genome, transcriptome, and proteome levels in many cancer types. The dysregulation of the circadian clock has important implications in glioma development and treatment [17–19, 21, 69]. With this study, we aimed to identify clock-regulated genes that play an important role in glioma development. We used publicly available datasets of LGG and GBM with single samples per patient, and multiple samples per patient to investigate the dysregulation in expression of clock-regulated genes, as well as to reconstruct progression patterns of mutations and phylogenetic trees in gliomas. Future studies that build upon our findings and include biological validation will be needed to investigate our hypothesis and strengthen our results.

We first explored the expression of 185 clock-regulated genes of interest in LGG and GBM, using single samples from the TCGA project. We found 4 differentially expressed genes of interest in LGG, and 45 in GBM, with 45 differentially expressed genes of interest separating tumour from normal samples in GBM. Among those genes, *CDK3*, is differentially expressed only in LGG for our dataset. *CDK3* belongs to the family of cyclin-dependent kinases, and is involved in regulating cell cycle progression [70]. It was previously reported to be overexpressed in GBM tissues and cell lines, where its overexpression was shown to promote cell proliferation and colony formation [65]. In our analysis, we identified overexpression of *CDK3* in LGG tumours compared to the normal tissue. Three additional differentially expressed genes identified by our analysis in LGG are *KIAA0101*, *MYC* and *HLF*. As previously reported, the expression of *KIAA0101* increases for higher tumour grades [63, 64]. The expression of *MYC* has also been shown to correlate with glioma grade [66]. Our results support previous studies which reported lower expression of *MYC* in normal vs. tumour tissue. However, our dataset does not show a clear trend of an increased *MYC* expression with an increase in tumour grade. Although the higher expression of *MYC* is associated with the higher-grade gliomas, our survival analysis reports better prognosis with higher *MYC* expression, when stratifying for tumour grade. *MYC* expression was not found to significantly contribute to survival when stratifying for IDH mutation status. The reason could be that, within each tumour grade group, higher expression of MYC correlates with better prognosis. A previous study, which explored the effect of expression of MYC proteins on survival of GBM patients, found no relationship between MYC expression and survival [71]. Our data shows the downregulation of *MYC* in the GBM samples compared to LGG. *CLOCK/BMAL* is known to regulate the cell cycle checkpoint regulators *MYC* (G1/S) and *WEE1* (G2/M) via the repression of *MYC* expression and activation of *WEE1* [72–74]. We observe an upregulation of *BMAL1* in GBM, which correlates to lower survival in these patients, associated to a decrease in *MYC* and increase in *WEE1* expression in GBM, in agreement with previous reports. According to the results of Dong et al. [20], *MYC* downregulation in GBM might indicate a high proportion of glioma stem cells in tumour samples. Considering the relevant role of *MYC* in the proliferation and survival of glioma stem cells [75], Dong et al. [20] speculate that in GBM, *MYC* and *BMAL1/CLOCK* cooperate to maintain the state of stemness, which would be in line with our results. Overexpression of *HLF* was previously reported to inhibit proliferation, invasion, and colony formation in human GBM cells [67]. In our analysis, *HLF* is downregulated in higher grade tumours. The majority of differentially expressed genes in GBM were upregulated (33/45). Interestingly, the set of downregulated genes in GBM contains three core-clock genes, *RORB*, *NR1D1* and *CRY2*. *NR1D1* and *CRY2* show a lower expression in higher grade gliomas. We further explored the effect of 45 differentially expressed genes and core-clock genes on patient survival. When stratifying for tumour grade, 17 genes were found to have a significant effect survival, of which four core-clock genes (*BMAL1/2*, *CRY2* and *RORC*). Our analysis indicates that low expression of *BMAL1/2* and *RORC* is associated with better prognosis, while *CRY2* shows the opposite trend. The association of high *CRY2* expression in GBM and better prognosis has been previously reported by Dong et al. [20]. The association of low *RORC* expression and better survival was also reported [76]. Low expression of *BMAL1* was previously associated with suppressed cell invasion, which supports our results [44]. The association between the expression of 8 other genes, namely, *BTRC* [77], *CALU* [78], *CHEK1* [79], *DTL* [80], *EEF1A1* [81], *KIAA0101* [63, 64], *MELK* [82], *PCNA* [83] and GBM, is in line with previously reported results. *FANCD2* expression was found to be correlated with glioma grade [84], and high expression was found to predict worse survival in lung adenocarcinoma [85]. *PTBP* promotes cell migration and differentiation in glioma cell lines [86], which agrees with our result that its lower expression leads to more favourable outcomes. High expression of *WEE1* has previously been associated with better prognosis [87], which contradicts our observation. However, the role of *WEE1* in glioma was also found to be connected to the *MGMT* status [87], which was not considered in our analysis.

We further explored the timing of mutations affecting genes of interest in the context of tumour progression by reconstructing progression patterns of mutations in genes of interest in LGG and GBM, and focusing on the clock-regulated drivers in glioma. Our results revealed that in LGG, an early small-scale mutation (SSM) in *TP53*, present in 53% of patients, is followed by either the loss of *TERT* (in 10% of patients) or the loss of *APC* (9% of patients). The loss of *HIF1A* is an early event, present in 21% of patients and occurring after the loss of *MGMT* (in 32% of patients). The loss of *TP53* and SSM in *APC* occur late in the LGG progression. On the contrary, in GBM, the loss of *TP53* occurs earlier in 17% of patients, and the losses of *TERT* and *APC* later, in 9% of patients. When considering multi-region data of LGG, we observe rare early clonal gains and losses of *APC, HIF1A* and *TP53.* Whole-genome doubling (WGD) events are present exclusively as subclonal events, and *APC*, *TERT*, *HIF1A* and *TP53* experience both late subclonal gains and losses, but with a comparably higher frequency. This might point to a role of the four clock regulated driver genes in the subclonal diversification and intra-tumour heterogeneity of LGG. Since our data is based on a historical data set, the actual WHO classification is not incorporated in the glioma gradings. The hallmark of the new WHO classification with its molecular distinction in the first place between IDH-wild type and IDH-mutant gliomas, and the following classification of all IDH wild type tumors as grade IV may lead to a shift from historic low grade (WHO II and III) to GBM grade IV, in our cohort. But nevertheless our key points related to circadian dysregulation still remain valid.

Our data consists of samples for one single timepoint, to overcome this limitation, we examined the time-dependent expression of the four potentially clock-regulated glioma drivers *APC*, *HIF1A*, *TERT* and *TP53* in HCT116 WT, and in the associated *BMAL1*, *PER2* and *NR1D1* KO cells. Their altered expression in the core-clock knockouts, as well the differential expression between each knockout and the WT cells is consistent with a regulation of these genes by clock elements. The downregulation observed in clock KO cells might be related to the potential role of the core clock in suppressing the cancer development (and/or progression) This is the case for example for the tumor suppressor *TP53* [88], which is downregulated in *BMAL1* and *PER2* KOs. *TERT* plays a role in the activity of telomerase [89] and *HIF1A* in the cellular response to hypoxia [90], promoting tumour growth. The overexpression of *TERT* and *HIF1A* in clock KO cells points indeed to their clock-regulated expression and further emphasizes the importance of the clock in the cellular response to tumour promoting conditions.

We also used data for which sampling time is unknown. TCGA data for GBM and LGG does not contain information about sampling times, which would be beneficial to fully explore the circadian dysregulation in gliomas. Only the subset of the LGG-JPN dataset, where multiple samples were taken during a single surgery, contains samples that were taken at the same time. Still, these datasets do not contain samples corresponding to different time points throughout a day. Although having such data would be advantageous, we still used the already existing data to explore an overall expression and place different mutation types in clock-regulated genes in perspective, based on the their time of emergence. In future studies, it would be beneficial to also include time-course data to study the extent of circadian dysregulation in gliomas.

Overall, our analysis suggests that the evaluated set of genes plays an important role in the development and progression of glial tumors. Further studies are needed to evaluate whether a manipulation of these genes via stimulation or blocking of their corresponding gene produts may have clinical impact in glial tumors.

## Conclusion

In summary, more information regarding the molecular heterogeneity of gliomas brings step-by-step light into the process of tumour progression and resistance development. In the future new potential therapeutic targets i.e. clock network genes may lead to outcome improvement for patients.

## Electronic supplementary material

Below is the link to the electronic supplementary material.

Supplementary Material 1

Supplementary Material 2

## Data Availability

The datasets analysed during the current study are available in the TCGA repository (https://portal.gdc.cancer.gov/), and European Genome-phenome Archive (EGA), accession code EGAS00001001044.

## Abbreviations

ADAM17: ADAM Metallopeptidase Domain 17 AKT3 - AKT serine/threonine kinase 3
APC: Adenomatous Polyposis Coli
BMAL1/2: Aryl hydrocarbon receptor nuclear translocator like 1/2
BTRC: Beta-transducin repeat containing E3 ubiquitin protein ligase
CALU: Calumenin
CAPRI: CAncer PRogression Inference
CCDC26: Coiled-Coil Domain Containing 26
CCK: Cholecystokinin
CCN: Core clock network
CDKN1A/2A/2B: Cyclin Dependent Kinase Inhibitor 1 A/2A/2B
CDKAL1: CDK5 regulatory subunit associated protein 1 like 1
CDK3: Cyclin Dependent Kinase 3
CDT1: Chromatin licensing and DNA replication factor 1
CHEK1/2: Checkpoint kinase ½
CLOCK: Clock circadian regulator
CNA: Copy number alteration
COSMIC: Catalogue of Somatic Mutations in Cancer
CRY1/2: Cryptochrome circadian regulator 1
DBP: D-box binding PAR bZIP transcription factor
DTL: Denticleless E3 ubiquitin protein ligase homolog
ECCN: Extended core clock network
EEF1A1: Eukaryotic translation elongation factor 1 alpha 1
EGA: European Genome-phenome Archive
EGFR: Epidermal growth factor receptor
EZH2: Enhancer of zeste 2 polycomb repressive complex 2 subunit
FANCD2: FA complementation group D2
GADD45A: Growth arrest and DNA damage inducible alpha
GEPIA2: Gene Expression Profiling Interactive Analysis
GNB2L1: Receptor for activated C kinase 1 (RACK)
GSEA: Gene set enrichment analysis
GBM: Glioblastoma multiforme
GPT: Glutamic-pyruvic transaminase
GRIN2C: Glutamate ionotropic receptor NMDA type subunit 2 C
GTEx: Genotype-Tissue Expression project
HBP1: HMG-box transcription factor 1
HDAC1: Histone deacetylase 1
HIF1A/2A: Hypoxia inducible factor 1/2 subunit alpha
HLF: HLF transcription factor, PAR bZIP family member
H3F3A: H3.3 histone A
IDH1/2: Isocitrate dehydrogenase (NADP(+)) ½
IRF7: Interferon regulatory factor 7
JPN-LGG: Japanese low-grade gliomas
KIAA0101: PCNA clamp associated factor (PCLAF)
LGG: Low-grade glioma
MEDICC2: Minimum Event Distance for Intra-tumour Copy-number Comparisons 2
MELK: Maternal embryonic leucine zipper kinase
MGMT: O-6-methylguanine-DNA methyltransferase
MYC: MYC proto-oncogene, bHLH transcription factor
NME1: NME/NM23 nucleoside diphosphate kinase 1
NCRG: Network of clock-regulated genes
NFIL3: Nuclear factor, interleukin 3 regulated
NONO: Non-POU domain containing octamer binding
NPAS2: Neuronal PAS domain protein 2
NR1D1: Nuclear receptor subfamily 1 group D member 1 (REV-ERBA)
PCNA: Proliferating cell nuclear antigen
PER1-3: Period circadian regulator 1–3
PHLDB1: Pleckstrin homology like domain family B member 1
PPIA: Peptidylprolyl isomerase A
PTBP1: Polypyrimidine tract binding protein 1
RELA: RELA proto-oncogene, NF-kB subunit
REST: RE1 silencing transcription factor
RORB/C: RAR related orphan receptor B/C
RPL5: Ribosomal protein L5
SNP: Single nucleotide polymorphism
SSM: Small-scale mutation
TEF: TEF transcription factor, PAR bZIP family member
TERT: Telomerase reverse transcriptase
TGFB1: Transforming growth factor beta 1
TIMELESS: Timeless circadian regulator
TMM: Trimmed mean of M values
TP53: Tumor protein p53
WEE1: WEE1 G2 checkpoint kinase
WES: Whole exome sequencing
WGD: Whole-genome doubling
WHO: World Health Organization
