# Supplementary material for: Differential expression of the circadian clock network correlates with tumour progression in gliomas
Source: BMC Med Genomics. 2023 Jul 3;16:154. doi: 10.1186/s12920-023-01585-w (PMC10316603; doi:10.1186/s12920-023-01585-w)
Supplement: Supplementary file 2 — Supplementary Material 2 [file 12920_2023_1585_MOESM2_ESM.docx]

**
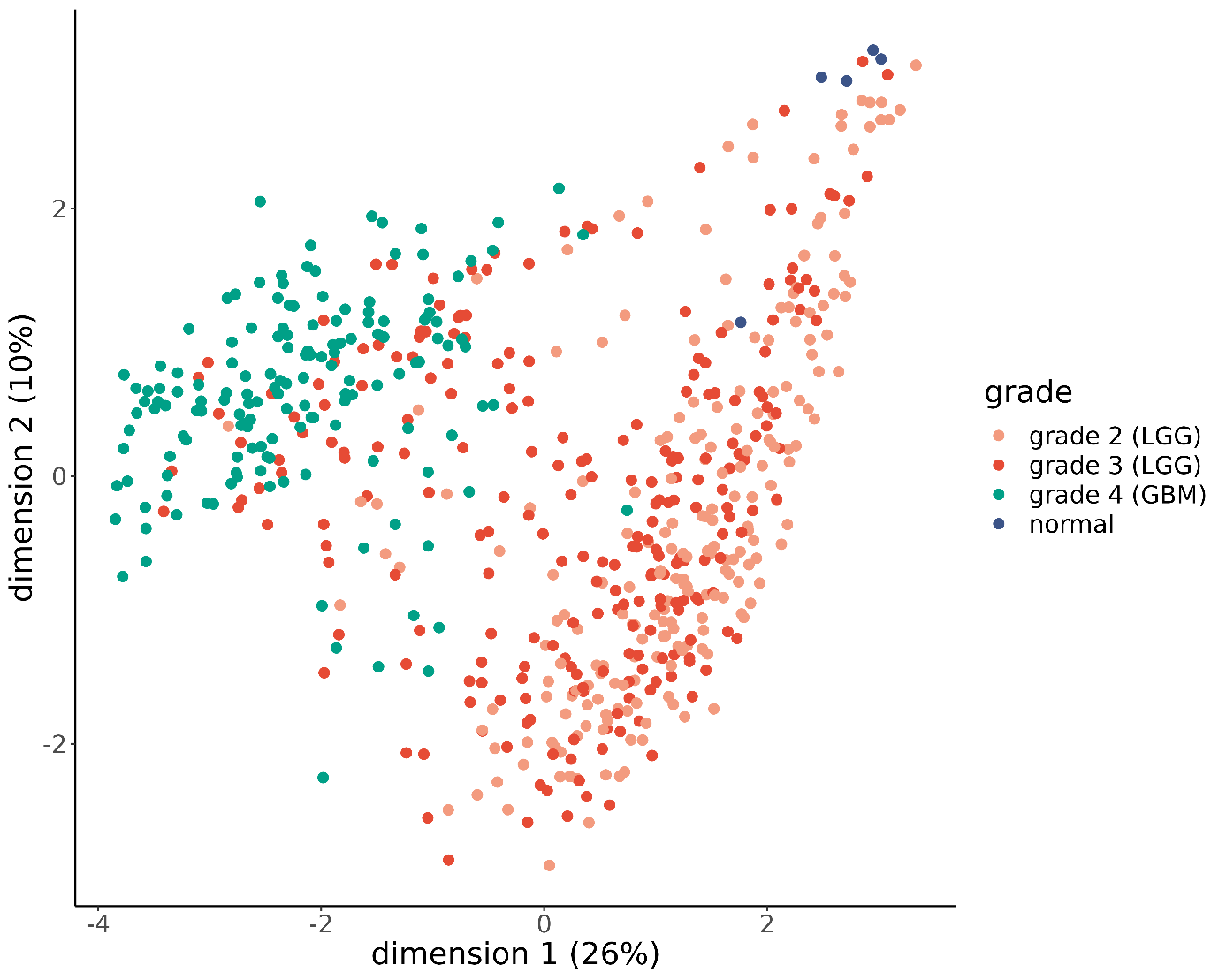
**

**Supplementary Figure 1.** Unsupervised clustering of patient samples based on the log-fold change in TCGA-LGG and TCGA-GBM dataset. Unsupervised clustering of 631 (216 grade 2 – LGG, 241 grade 3 – LGG, 169 grade 4 – GBM, 5 normal) patient samples. Grade 2 samples are colored orange, grade 3 samples red, grade 4 samples green and normal samples blue. Dimensions 1 and 2 explain 36% of the variation in the data.


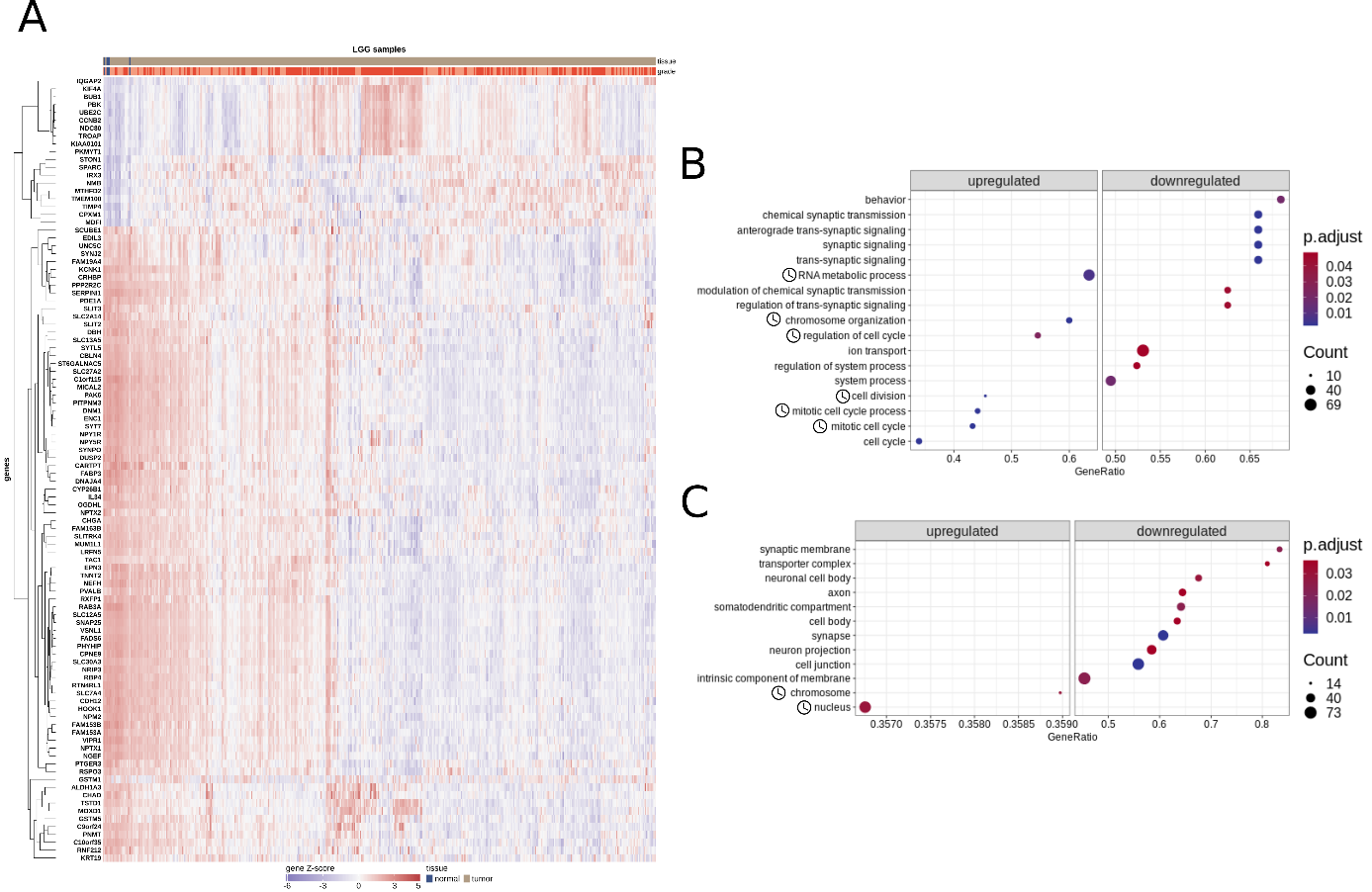


**Supplementary Figure 2.** Differentially expressed genes in TCGA-LGG. **A** Hierarchical clustering of top 100 differentially expressed genes. Normal samples are marked in blue and tumor samples in brown. Grade 2 samples are shown in orange and grade 3 in red. **B** GSEA analysis for biological process enriched for differentially expressed genes of interest. The smallest term contains 10, and the largest set 69 core genes. Terms that contain differentially expressed clock genes are marked with a clock. **C** GO terms for cellular component enriched for differentially expressed genes of interest. The smallest term contains 14 and the largest set 73 genes. Terms containing genes of interest are marked with a clock.


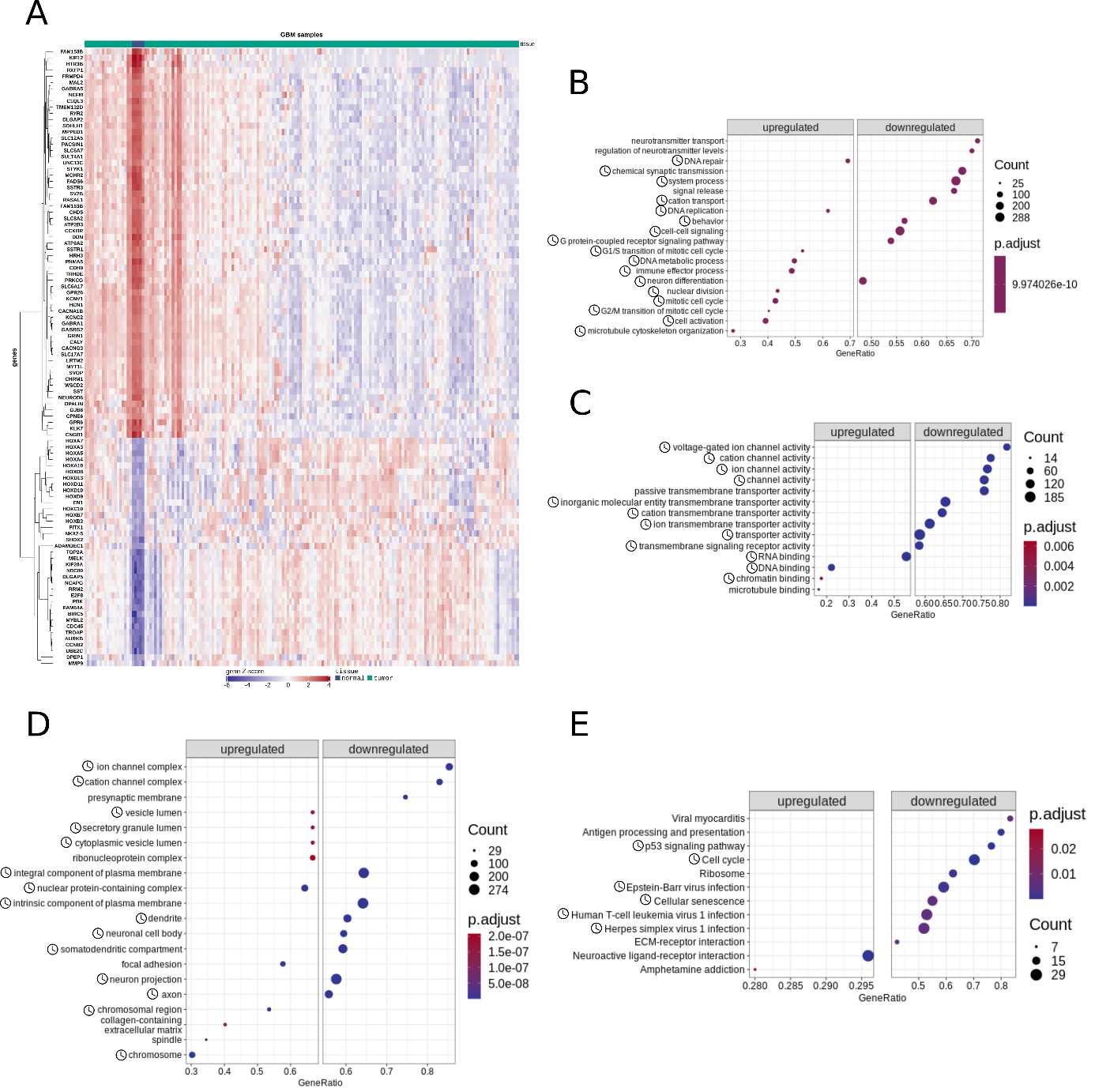


**Supplementary Figure 3.** Differentially expressed genes in TCGA-GBM. **A** Hierarchical clustering of top 100 differentially expressed genes. Normal samples are marked in blue and tumor samples in brown. **B** 10 most enriched GO terms for the biological process ontology. The smallest term contains 25, and the largest 288 genes. **C** 10 most enriched GO terms for the molecular function ontology. The smallest set contains 14, and the largest set 185 genes. **D** 10 most enriched GO terms for the cellular component ontology. The smallest term contains 29, and the largest term 274 genes. **E** Top 10 enriched KEGG pathways. The smallest set contains 7, and the largest set 29 genes. In **B**, **C**, **D** and **E**, the terms and pathways that contain differentially expressed genes of interest are marked with a clock.


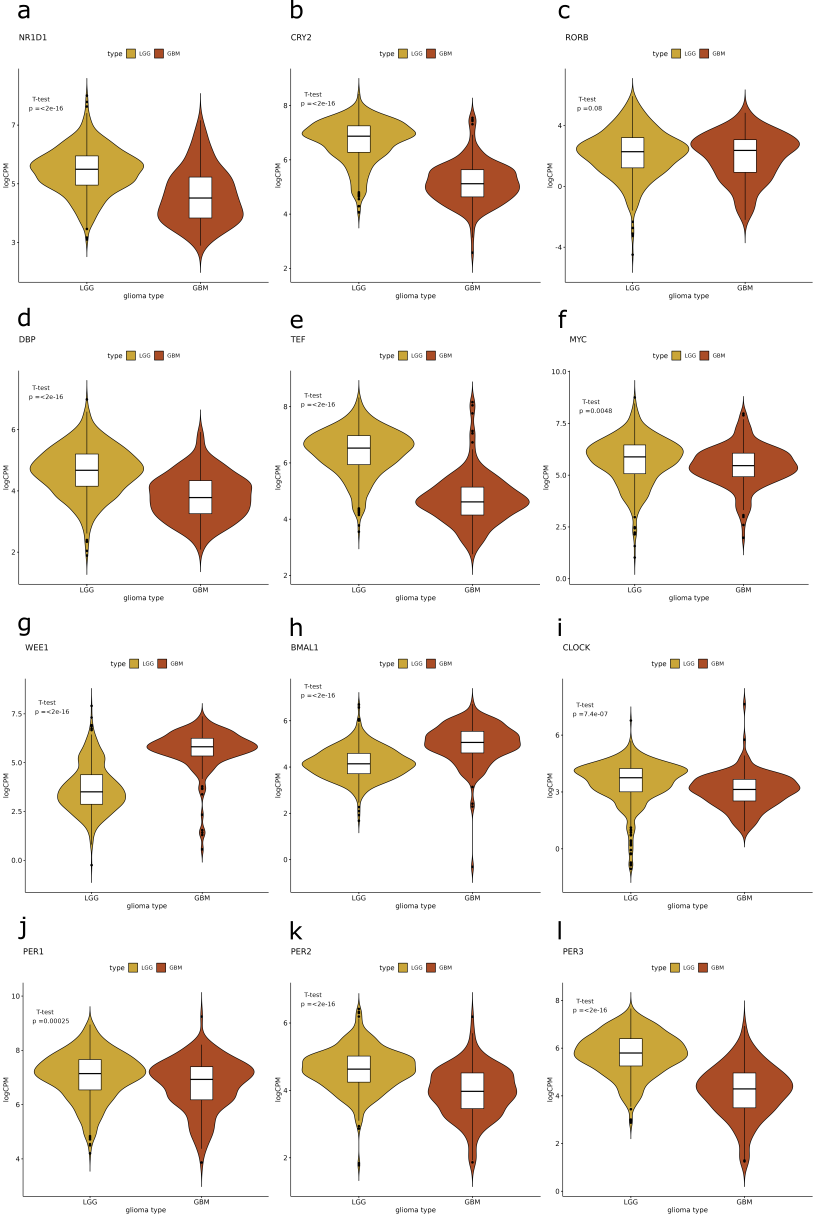


**Supplementary Figure 4.** Clock genes and cell cycle check point genes are differentially expressed in GBM. Expression of *NR1D1, CRY2, RORB, DBP, TEF, MYC, WEE1, BMAL1, CLOCK,* and *PER1-3* for 685 patients in LGG and GBM.


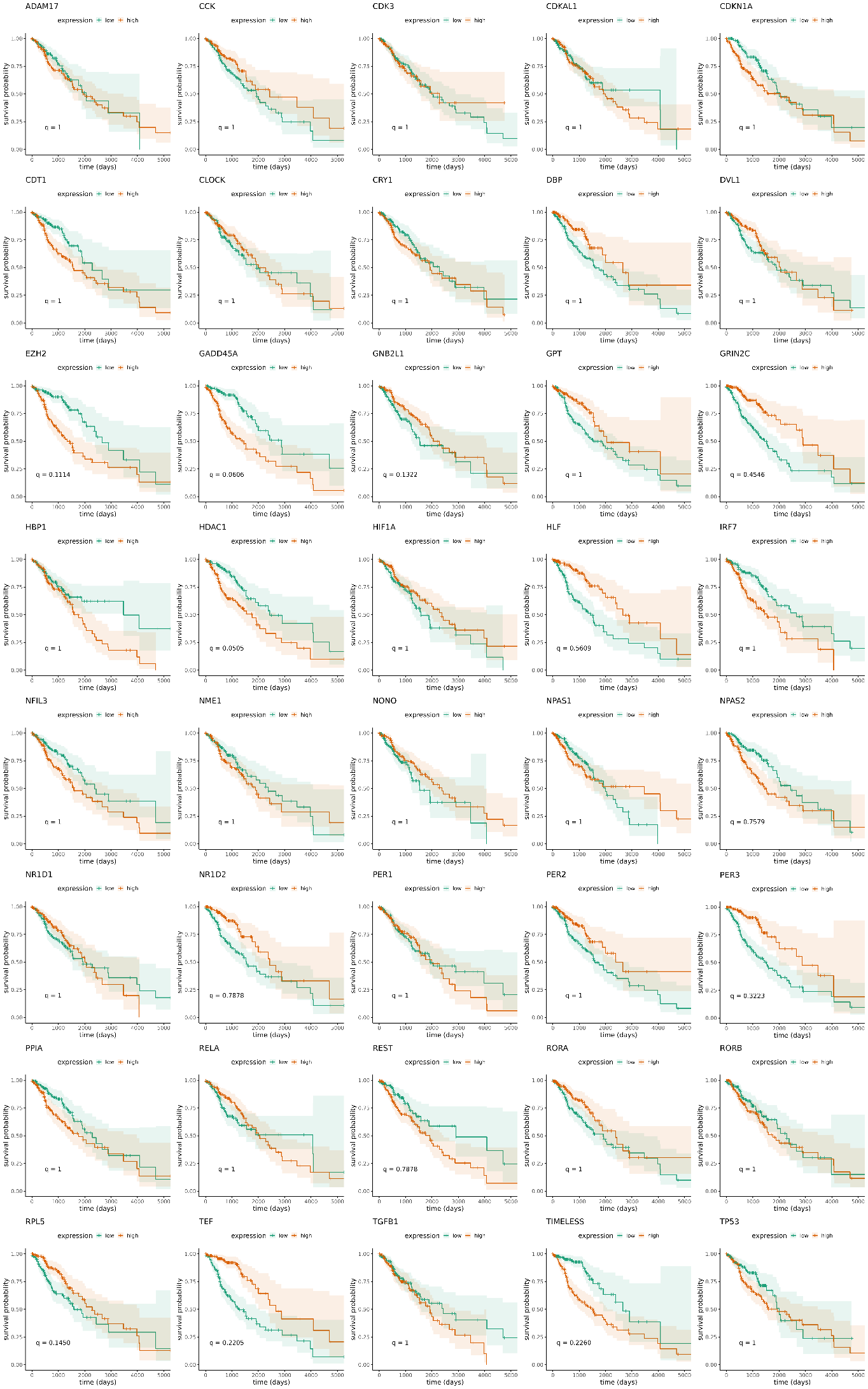


**Supplementary Figure 5.** Survival analysis of differentially expressed genes of interest and CCN genes, stratification based on tumor grade. Kaplan-Meier curves for 40 genes with non-significant effect on survival (*q* >= 0.05). Green shows low and red high expression.


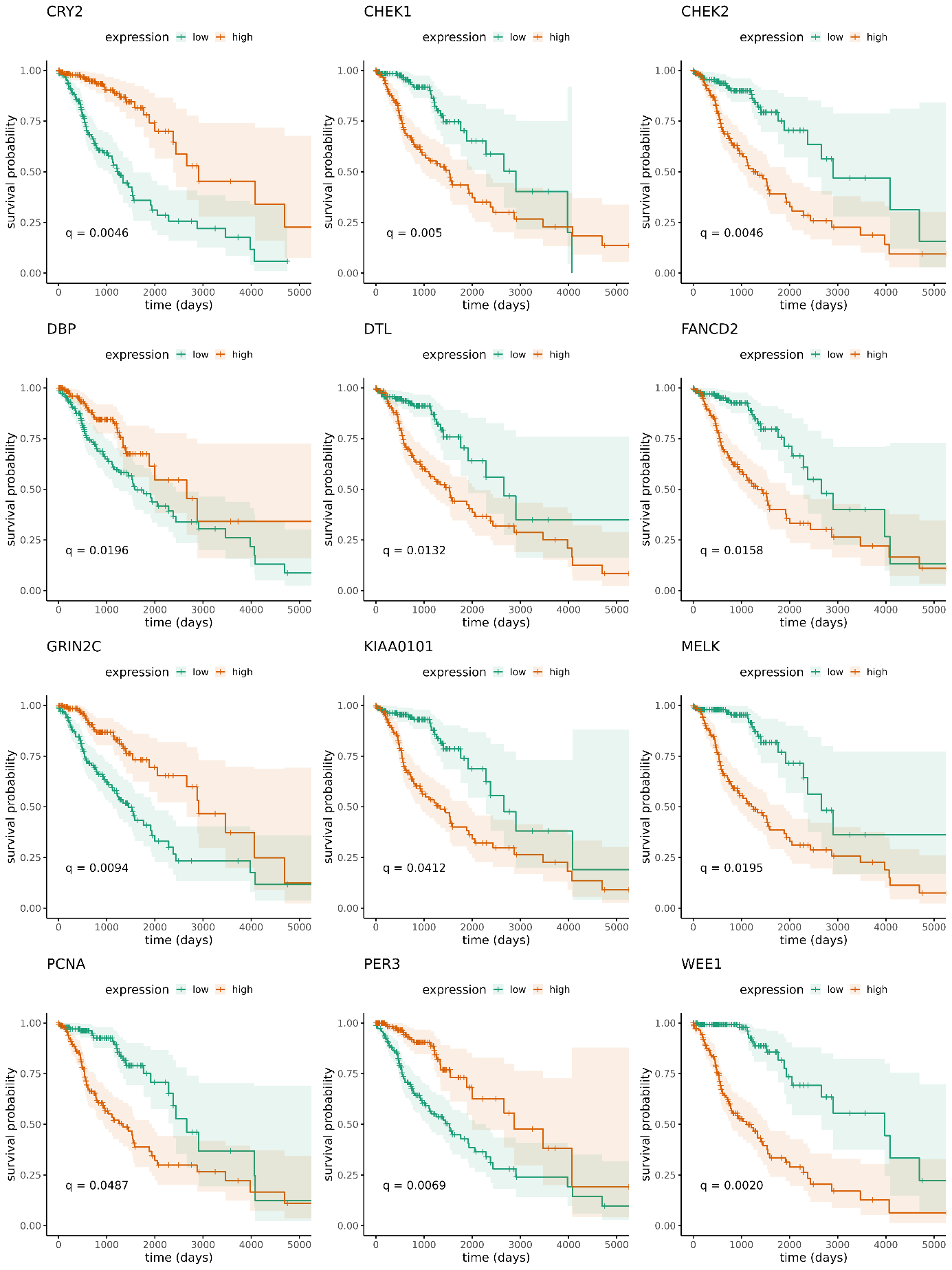


**Supplementary Figure 6.** Survival analysis of differentially expressed genes of interest and CCN genes, stratification based on IDH mutation status. Kaplan-Meier curves for 12 genes with a significant effect on survival (*q* < 0.05). Green shows low and red high expression.


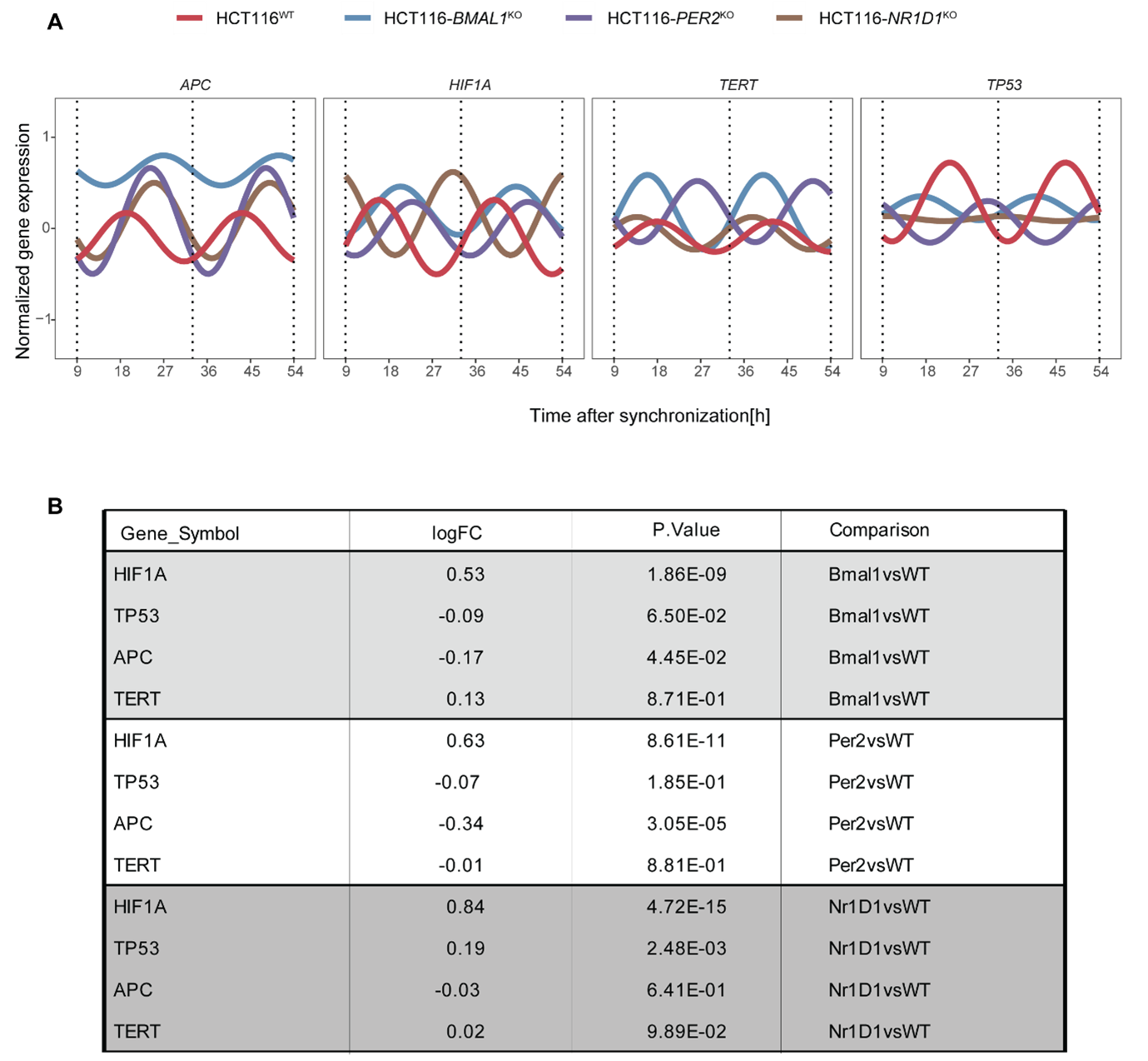


**Supplementary Figure 7.** Impact of core-clock alterations in selected tumor suppressor genes. **A** 24h time-dependent expression of APC, HIF1A, TERT and TP53 in HCT116 WT and KO cells. The time series expression is represented with 24h harmonic regression curves in HCT116 and -derived KO cells (HCT116 WT, HCT116-BMAL1KO, HCT116-PER2KO, HCT116-NR1D1KO). **B** Differential expression analysis for APC, HIF1A, TERT and TP53 in each KO vs the WT cell line.

**
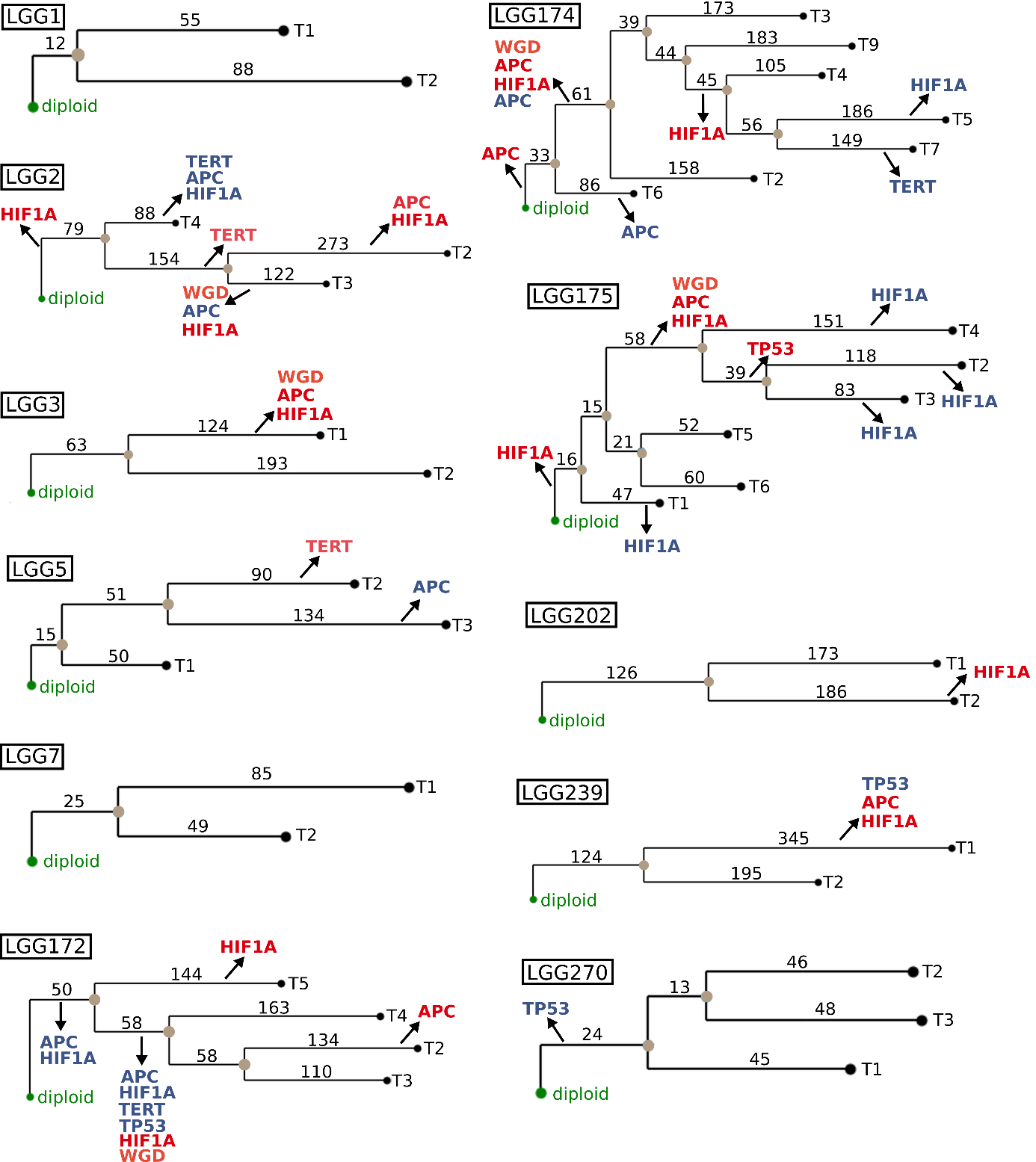
**

**Supplementary Figure 8.** Evolutionary trees for 11 JPN-LGG patients. Evolutionary trees for 11 patients from JPN-LGG dataset. Diploid (normal) node is marked in green, internal nodes in brown and leaves in black. Genes with gains are indicated in red and with losses in blue. WGD is indicated in orange. Numbers on branches indicate the distance of adjacent nodes in terms of number of events of gains and losses. Patients LGG1, LGG2, LGG3, LGG5, LGG7, LGG202, LGG239 and LGG270 belong to the group of patients with multiple timepoints, and patients LGG172, LGG174, LGG175 to the group of patients with multiple regions.
